# Global burden and temporal trend of thyroid cancer associated with high BMI from 1990 to 2021

**DOI:** 10.1371/journal.pone.0343394

**Published:** 2026-03-20

**Authors:** Dan Pan, Ying-Xiu Diao, Yu-Xiang Pan, Zi-Hang Ai, Qing-Yang Liu, Zan-Bin Li

**Affiliations:** 1 Department of Rehabilitation Medicine, First Affiliated Hospital of Gannan Medical University, Ganzhou, Jiangxi, China; 2 School of Rehabilitation Medicine, Gannan Medical University, Ganzhou, Jiangxi, China; 3 Department of Rehabilitation Medicine, Gannan Healthcare Vocational College, Ganzhou, Jiangxi, China; 4 Department of Thyroid and Hernia Surgery, First Affiliated Hospital of Gannan Medical University, Ganzhou, Jiangxi, China; Texas Tech University Health Science, Lubbock, UNITED STATES OF AMERICA

## Abstract

The global incidence of thyroid cancer (TC) has risen markedly, ranking as the tenth most common malignancy in 2020. Simultaneously, obesity now affects over one billion individuals worldwide, with its prevalence more than doubling since 1990. Emerging evidence suggests a significant association between high body mass index (BMI) and elevated TC risk, potentially mediated by mechanisms such as chronic inflammation and insulin resistance. This study aims to evaluate the global burden of thyroid cancer attributable to high BMI (TC-HBMI) from 1990 to 2021, examine temporal trends, and project future burden through 2036. We utilized data from the Global Burden of Disease Study 2021 to assess deaths, disability-adjusted life years (DALYs), and age-standardized rates (ASRs). Temporal trends were examined using estimated annual percentage change (EAPC), joinpoint regression, and decomposition analysis. Inequality and future burden assessments were conducted through slope and concentration indices, frontier analysis, and Bayesian age-period-cohort modeling.

## 1. Introduction

Over recent decades, a significant global rise in the incidence of thyroid cancer (TC) has been observed. Notably, the rate at which TC incidence has increased surpasses that of many other types of malignant tumors [1]. In 2020, approximately 586,000 individuals worldwide were diagnosed with TC, positioning it as the tenth most common cancer globally [2]. The considerable number of cases and the projected future increase underscore the growing public health significance of TC. In 2019, TC accounted for 1231.84 thousand disability-adjusted life years (DALYs) globally [3,4]. DALYs serve as a comprehensive metric to quantify the overall burden of disease, taking into account both years of life lost due to premature mortality and years lived with disability. The long-term management and potential complications associated with TC contribute to the morbidity experienced by patients, affecting their overall quality of life. Recent statistics from the World Health Organization (WHO) and other organizations indicate that over one billion people worldwide are now living with obesity. In 2022, approximately 43% of adults were overweight, and 16% were obese, with rates having more than doubled since 1990 [5]. The escalating global incidence of TC coupled with the widespread prevalence of obesity underscores the potential for a substantial public health impact if a causal relationship exists between these two conditions.

Several established and emerging risk factors have been implicated in the development of TC. Known risk factors include exposure to radiation, particularly during childhood, a family history of TC, increasing age (especially over 40 years), and female gender [6]. Certain genetic conditions also increase the risk of specific types of TC. In addition to these well-established factors, research has explored the potential role of lifestyle changes, environmental pollutants, dietary factors such as iodine intake and nitrates, reproductive factors, and obesity in thyroid carcinogenesis.

Among the emerging risk factors, the association between adiposity, as measured by body-mass index (BMI), and the risk of TC has garnered increasing attention. Several meta-analyses have suggested a statistically significant association between higher BMI (both overweight and obesity) and an increased risk of developing TC [7–11]. Furthermore, high BMI has been found to contribute significantly to the burden of TC as measured by DALYs and mortality, particularly in the Middle East and North Africa region [12]. The impact of obesity/overweight on TC extends beyond just the risk of developing the disease, potentially influencing its prognosis and treatment outcomes. Several studies have investigated the association between BMI and aggressive clinicopathological features of TC, such as tumor size, extrathyroidal extension, and lymph node metastasis [13]. The association between obesity/overweight and TC risk is likely mediated by a complex interplay of biological mechanisms. Chronic low-grade inflammation, a hallmark of obesity, is considered a key contributor [14]. Adipose tissue, in an obese state, functions as an endocrine organ, secreting various pro-inflammatory cytokines such as tumor necrosis factor-alpha (TNF-α) and interleukin-6 (IL-6) [15]. Insulin resistance and the consequent hyperinsulinemia, frequently observed in obese individuals, represent another significant pathway [9]. Insulin, beyond its role in glucose metabolism, can act as a growth factor, potentially stimulating the proliferation of thyroid cells and contributing to tumor development [16]. Furthermore, obesity is associated with dysregulation of adipokines, which are hormones secreted by adipose tissue. Other potential mechanisms linking obesity and TC include increased oxidative stress and hormonal changes, which are often associated with excess adiposity and can contribute to cellular damage and altered cell growth patterns [14].

Although a recent study have utilized GBD 2021 to explore the burden of TC attributable to high BMI (TC-HBMI), the study primarily focus on global overview analyses [17]. In contrast, our study not only aim to provide a comprehensive overview of the global burden of TC attributable to high body-mass index from 1990 to 2021, but also employs a range of advanced statistical and analytical methods, including joinpoint regression, frontier analysis, and health inequality analysis, to comprehensively examine the trends of TC attributable to high BMI and the associated health inequalities across different levels of socioeconomic development worldwide. By analyzing the observed trends in TC incidence, prevalence, mortality, and DALYs during this period, with a specific focus on the contribution of high BMI, this study seeks to enhance our understanding of the relationship between this significant modifiable risk factor and the global epidemiology of TC. Furthermore, we will present forecasted trends up to 2036, taking into consideration the potential impact of high BMI on the future burden of the disease.

## 2. Methods

### 2.1. Data sources and definitions

Our study reported on the disease burden of TC attributable to high BMI, analyzing various age categories (male and female) in 204 countries, 21 regions and 5 SDI regions in data of 2021 [18,19]. The procedural specifics of the GBD have been outlined in previous works. Access the information and procedures for GBD 2021 via the GBD Results Tool on the Global Health Data Exchange website (http://ghdx.healthdata.org/gbd-results-tool). Within the context of the GBD, high BMI for adults (ages 20 and older) is defined as BMI greater than 20–23 kg/m2. High BMI for children (ages 2–19) is defined as being overweight or obese based on International Obesity Task Force standards(https://www.healthdata.org/research-analysis/diseases-injuries-risks/factsheets/2021-high-body-mass-index-level-2-risk). The definition of TC is malignant neoplasms of the thyroid; ICD-10 code C73 (https://www.healthdata.org/research-analysis/diseases-injuries-risks/factsheets/2021-thyroid-cancer-level-3-disease).

### 2.2. Estimation of attributable burden

The proportion of thyroid cancer burden attributable to high BMI was derived from the Population Attributable Fraction (PAF) estimated by the GBD 2021 comparative risk assessment framework. PAF values are calculated by IHME using exposure distributions of BMI by age, sex, year, and location, together with meta-analytic relative risks for thyroid cancer (ICD-10 C73). The GBD model incorporates adjustment for correlations among all included risk factors within its joint attribution algorithms; however, it does not explicitly control for non‑GBD factors such as radiation exposure or genetic predisposition. Therefore, our analyses represent the independent contribution of high BMI among the metabolic risk factors considered in GBD.

### 2.3. Joinpoint regression analysis

This study employs joinpoint regression analysis to evaluate the changing trends of the burden associated with TC-HBMI, particularly the annual changes in ASDR and ASMR. We processed the TC-HBMI -related data from 1990 to 2021 using Joinpoint software (version 4.9.1.0) by identifying the joinpoints in the data, segmenting different time periods, and calculating the Average Annual Percentage Change (AAPC) within each period [20]. For each determined time period, we calculated the AAPC to reveal the changing trends of the burden of TC-HBMI across different time intervals. We investigated whether the fluctuations in different segments were statistically significant by comparing the AAPC to 0, with a statistically significant p-value defined as < 0.05.

### 2.4. Decomposition analysis

This study explores the changes in deaths and DALYs associated with TC-HBMI from 1990 to 2021 through decomposition analysis, with particular attention to the effects of population changes, aging, and epidemiological changes on the increase or decrease of deaths and DALYs.

### 2.5. Frontier analysis

To further explore the spatial and temporal trends of the TC-HBMI burden, we employed a frontier analysis method. This analysis, combined with the Sociodemographic Index (SDI), assesses the potential for further reductions in disease burden based on the current socio-economic development level of each country, revealing the relationship between SDI and TC-HBMI burden. The frontier analysis helps to identify the gap between the actual burden and the potential lower burden that could be achieved under the current socio-economic conditions.

### 2.6. SDI correlation analysis

The GBD 2021 study uses the Socio-Demographic Index (SDI) as a composite measure of socio-economic status closely related to health outcomes. SDI combines per capita income, educational attainment, and total fertility rate (TFR) to assess the socio-demographic development of a country. According to the GBD 2021 study, SDI is categorized into five levels: high SDI (>0.81), high-middle SDI (0.70–0.81), middle SDI (0.61–0.69), low-middle SDI (0.46–0.60), and low SDI (<0.46). This study explores the relationship between the burden of TC-HBMI and SDI through correlation analysis, aiming to reveal the impact of socioeconomic development on disease burden. We use the Pearson correlation coefficient to assess the correlation between SDI and the burden of TC-HBMI, with specific calculations involving the identification of SDI values for each country and region alongside their corresponding deaths and DALYs data.

### 2.7. Cross-country inequality analysis

Cross-national health inequality analysis aims to assess the absolute and relative inequalities in the burden of TC-HBMI among different countries and regions. This study quantifies the differences in DALYs associated with TC-HBMI across countries with varying levels of socioeconomic development by calculating the Slope Index of Inequality (SII) and the Concentration Index [21]. The SII reflects the degree of linear inequality in the burden of TC-HBMI between countries, with higher values indicating more severe inequality. The Concentration Index is used to assess the concentration of ASMR and ASDR among countries with different income levels. Specifically, we rank the ASMRs and ASDRs of each country and calculate their distribution across different SDI groups to obtain the Concentration Index value, which ranges from −1–1, with values closer to 1 indicating that the burden is more concentrated in low-SDI countries.

### 2.8. Bayesian age-period-cohort analysis

BAPC forecasting analysis is a powerful statistical method used to assess and predict changes in TC-HBMI across different ages, time periods, and birth cohorts. This study analyzes TC-HBMI related data collected from 1990 to 2021 using the BAPC model. Based on the model output, we predict the trend of deaths, DALYs, ASDR and ASMR associated with TC-HBMI by the year 2036. The BAPC projections were based on historical GBD 2021 trends and assume a continuation of existing trajectories in high BMI exposure and thyroid cancer outcomes. No adjustments were made for potential future changes in obesity prevalence or the impact of new interventions; thus, the forecasts should be interpreted as baseline estimates.

### 2.9. Statistics

This study employs various statistical methods to analyze the disease burden of TC-HBMI, ensuring the accuracy and reliability of the results. All data processing and statistical analyses were conducted using R language (version 4.3.0) and the corresponding statistical packages. All statistical tests were two-sided, and P values less than 0.05 were considered statistically significant.

## 3. Results

### 3.1. Global trends in the burden of TC-HBMI from 1990 to 2021

**Fig 1:**
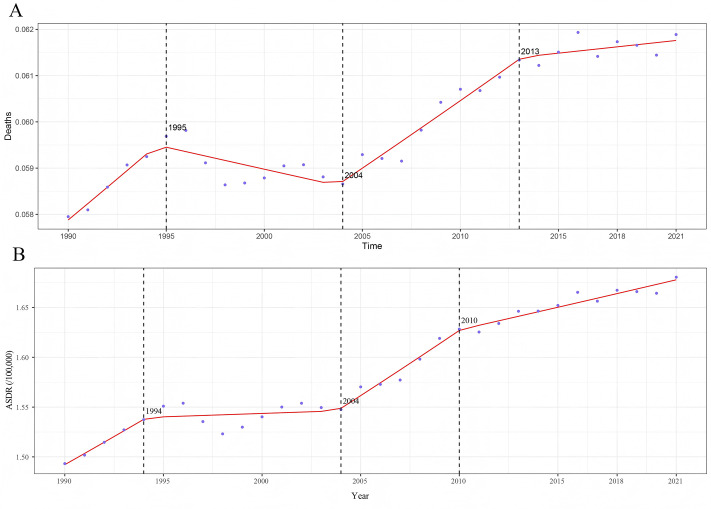
Trends of ASMR and ASDR of TC-HBMI during 1990–2021. **(A)** join-point model of TC-HBMI mortality rate. **(B)** join-point model of TC-HBMI DALY rate. ASMR: Age-standardized mortality rate. ASDR: Age-standardized DALY rate. TC-HBMI: Thyroid cancer attributable to high body-mass index. DALY: Disability-adjusted life year.

Analysis of the GBD 2021 data reveals that in 2021, 11.72% of TC deaths (95% UI: 8.84, 14.57) and 11.62% of TC-HBMI DALYs (95% UI: 8.81, 14.47) were attributable to high BMI. This represents an increase from 10.03% (95% UI: 7.53, 12.74) and 9.55% (95% UI: 7.17, 12.12) in 1990, respectively (S1 Table). The segmental trends of mortality rates and DALY rates of TC-HBMI were divided using join-point analysis (Fig 1). The ASMR and ASDR of TC-HMBI significantly increased since 1990, with three major increases during 1990−1995, 2004–2013 and 2013–2021 (APC1990−1995 = 0.611%, APC 2004–2013 = 0.491%, APC2013−2021 = 0.074%), along with a period decrease during 1995−2004(APC = −0.161%). While the DALY rate remained increaing from 1990 to 2021. The highest APCs of mortality were found in 2004–2010 (APC = 0.824%).

In 2021, the ASMRs and ASDRs for TC-HBMI were 0.06 per 100,000 (95% UI:0.04,0.08) and 1.68 per 100,000 (95% UI:1.26,2.14), respectively. While death number and DALY number were 5254.93(3913.53,6652.52) and 144954.89(109229.87,184747.27), respectively. The burden of age-standardized rates of DALYs and death numbers for 1990 and 2021 across 204 countries worldwide is presented in S2 Table. From 1990 to 2021, ASMRs (EAPC: 0.20; 95% CI: 0.16,0.24) and ASDRs (EAPC: 0.38; 95% CI: 0.34, 0.41) showed significant increasing trends. Geographically, there is considerable heterogeneity in the trend of TC-HBMI. The highest annual increase in ASMR was observed in South Asia (+2.35%) and Southern Sub-Saharan Africa (+1.76%), followed by North Africa and Middle East (+1.53%) and Southeast Asia (+1.38%). A similar regional trend was seen for ASDR, the highest annual increase in ASDR was observed in South Asia (+2.33%) and Southern Sub-Saharan Africa (+1.74%), followed by Southeast Asia (+1.42%) and North Africa and Middle East (+1.41%). Conversely, the regions with the largest annual decrease in ASMR were Central Europe (−1.97%), Western Europe (−1.23%), and Southern Latin America (−0.50%). The greatest annual decrease in ASDR occurred in Central Europe (−2.03%), Western Europe (−1.03%), and Central Asia (−0.71%) (S3 Table). The EAPC of ASMR and ASDR of 1990–2021 across 204 countries worldwide is presented in S4 Table.

### 3.2. Global burden of TC-HBMI: Age, gender and SDI patterns

**Fig 2:**
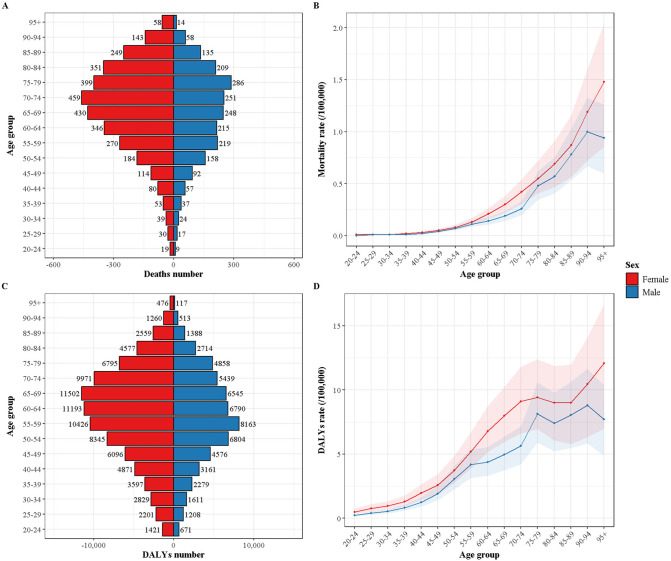
Age and gender specific numbers and rates of deaths and DALYs of TC-HBMI in 2021. **(A)** Death numbers by sex and age groups **(B)** Death rates by sex and age groups **(C)** DALYs numbers by sex and age groups **(D)** DALYs rates by sex and age groups. TC-HBMI: Thyroid cancer attributable to high body-mass index. DALY: Disability-adjusted life year.

Age and gender-specific patterns revealed that the deaths of TC-HBMI initially increased with age before declining, peaking in the 70–74 age group for females and the 75–79 age group for males (Fig 2A). And DALYs peaked at 65–69 age group for females and 55–59 age group for males (Fig 2B). The mortality rate in females continues to increase with advancing age, whereas in males, it peaks in the 90–95 age group. Additionally, the DALY rates in females increase with age, showing a gradual plateau in the 75–89 age group, followed by a significant rise. In males, the DALY rates peak in the 90–95 age group. Across all age groups, DALYs and ASR were higher in females than in males.

**Fig 3:**
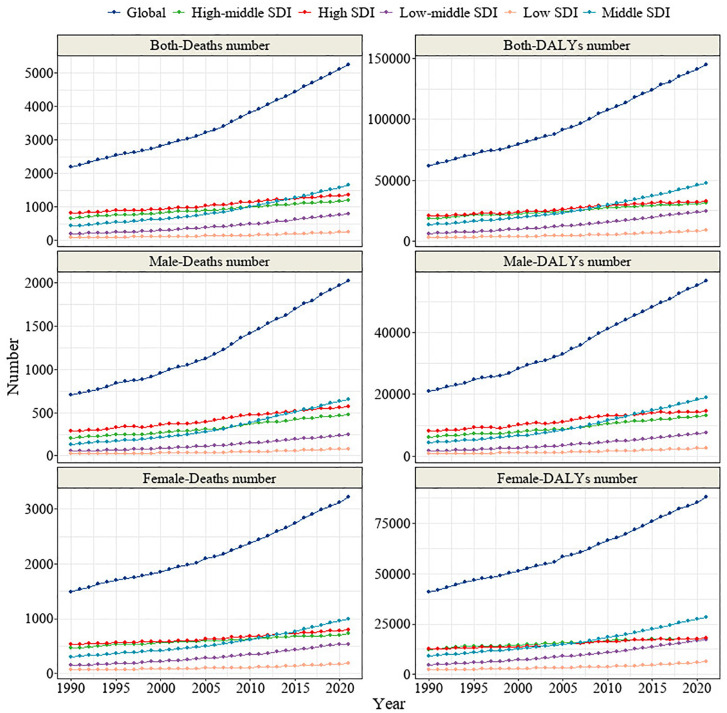
Trends in death and DALY numbers for TC-HBMI across five different SDI regions and two genders. TC-HBMI: Thyroid cancer attributable to high body-mass index. DALY: Disability-adjusted life year. SDI: Sociodemographic Index.

**Fig 4:**
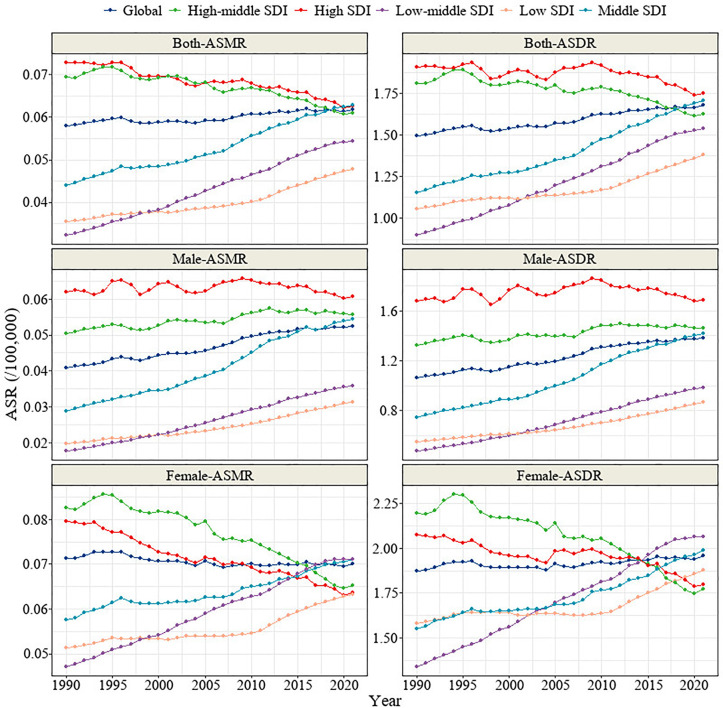
Trends in ASMR and ASDR for TC-HBMI across five different SDI regions and two genders. ASMR: Age-standardized mortality rate. ASDR: Age-standardized DALY rate.TC-HBMI: Thyroid cancer attributable to high body-mass index. DALY: Disability-adjusted life year. SDI: Sociodemographic Index.

Regarding the trends in mortality and DALY rates for TC-HBMI across five different SDI regions, all regions showed a consistent upward trend in both mortality and DALY rates. This upward trend was consistent across gender groups. Although high-SDI regions maintained higher absolute levels of mortality and DALYs, the rate of increase over time was significantly slower than in low- and middle‑SDI regions, suggesting a relative plateau in disease burden in more developed areas (Fig 3). In most regions (middle, low-middle, and low SDI regions), both the ASMR and ASDR exhibited an upward trend. However, there was heterogeneity in ASMR and ASDR trends between gender groups. In high-middle SDI regions, the ASMR and ASDR in the male group showed an increasing trend, while the female group showed a decreasing trend. In high-SDI regions, the male group displayed a stable trend in both ASMR and ASDR, while the female group showed a marked decline (Fig 4).

**Fig 5:**
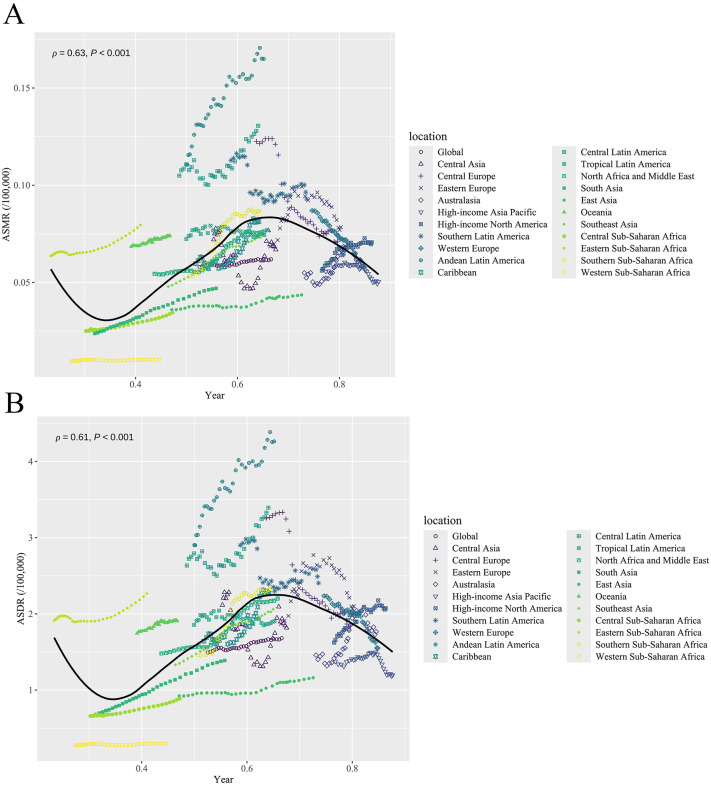
ASMR and ASDR of TC-HBMI across 21 Global Burden of Disease regions by Socio-Demographic Index, 1990–2021. **(A)** ASMR. **(B)** ASDR. ASMR: Age-standardized mortality rate. ASDR: Age-standardized DALY rate.

As SDI values increase, there is heterogeneity in the ASDR and ASMR of TC-HBMI across regions. Notably, Andean Latin America, Central Sub-Saharan Africa and Central Latin America have higher ASDR and ASMR of TC-HBMI, which are markedly higher than those in other regions with the same SDI value (Fig 5). Our correlation analysis shows that ASMR of TC-HBMI are significantly positively correlated with SDI (ρ = 0.63, p < 0.001). Similarly, the ASDR of TC-HBMI model shows a significant positive correlation with SDI (r = 0.61, p < 0.001).

**Fig 6:**
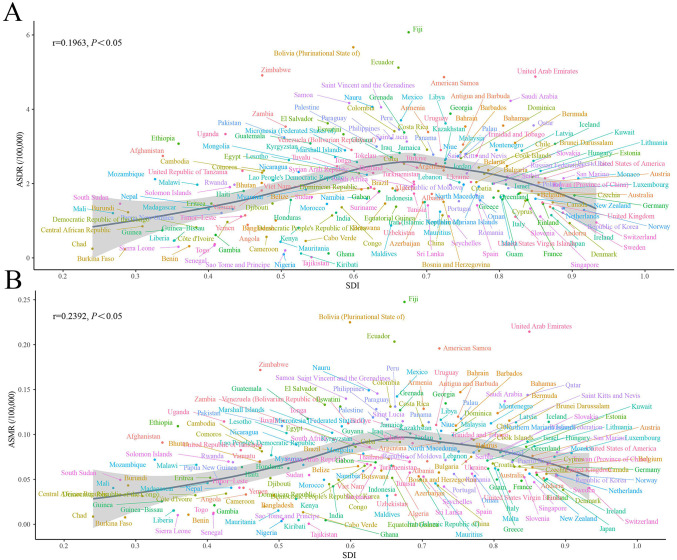
Age-standardized rates of mortality and DALYs across 204 countries and territories by Socio-Demographic Index in 2021. **(A)** Mortality. **(B)** DALYs. DALY: Disability-adjusted life year.

In our correlation analysis of 204 countries, ASMR was weakly yet significantly positively correlated with SDI (r = 0.1963, p < 0.05), and ASDR was weakly but significantly negatively correlated with SDI (r = –0.2392, p < 0.05) (Fig 6).

**Fig 7:**
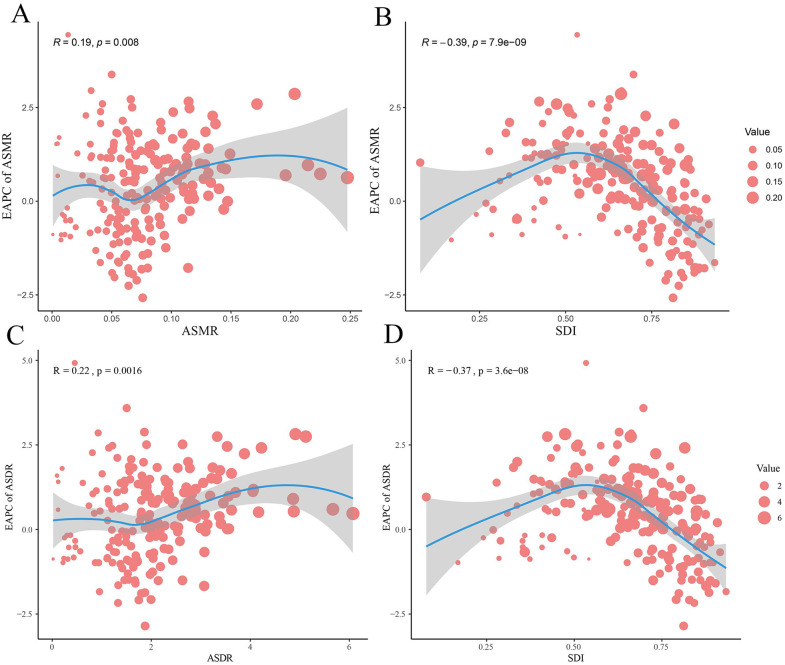
The correlation between EAPC of ASMR/ASDR and ASMR/ASDR and the correlation between EAPC of ASMR/ASDR and SDI. **(A)** Correlation between EAPC of ASMR and ASMR. **(B)** Correlation between EAPC of ASDR and ASDR. **(C)** Correlation between EAPC of ASMR and SDI. **(D)** Correlation between EAPC of ASDR and SDI. ASMR: Age-standardized mortality rate. ASDR: Age-standardized DALY rate. DALY: Disability-adjusted life year. EAPC: estimated annual percentage change. SDI: Sociodemographic Index.

In the correlation analysis of EAPC of ASMR and ASDR, a significant positive correlation between ASMR/ASDR and EAPC of ASMR/ASDR was observed, respectively (Fig 7A, 7C). While EAPC of ASMR/ASDR exhibits a significant negative correlation with SDI (Fig 7B, 7D).

### 3.3. Decomposition analysis of TC-HBMI trends

**Fig 8:**
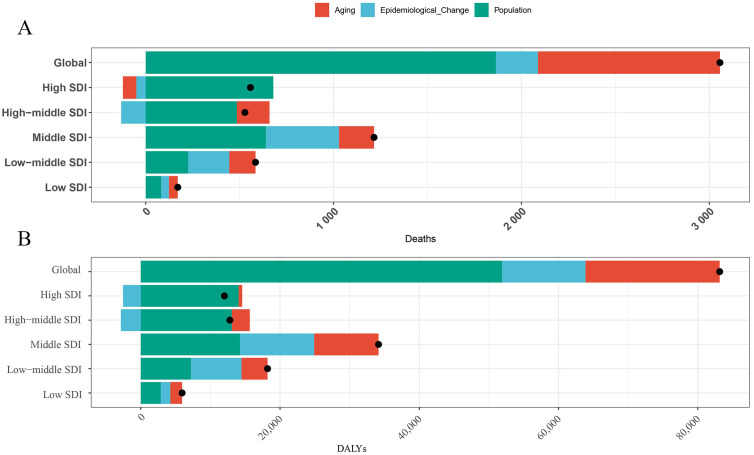
Decomposition analysis of the trends in deaths and DALYs of TC-HBMI from 1990 to 2021. TC-HBMI: Thyroid cancer attributable to high body-mass index. DALYs, disability-adjusted life years.

To investigate the impact of population growth, ageing and epidemiological changes on trends in deaths and DALYs of TC-HBMI from 1990 to 2021, a decomposition analysis was performed (Fig 8). The results indicate that the main influence on deaths of TC-HBMI is population change. Population change acts positively on TC-HBMI deaths in the 5 SDI regions. Secondly, the main influencing factor for DALYs of TC-HBMI was also population change, which showed a positive effect in all regions. Finally, deaths and DALYs of TC-HBMI in high-middle and high SDI regions are negatively affected by demographic change, and deaths of TC-HBMI in high SDI regions are also negatively affected by ageing.

### 3.4. Health inequality analysis

**Fig 9:**
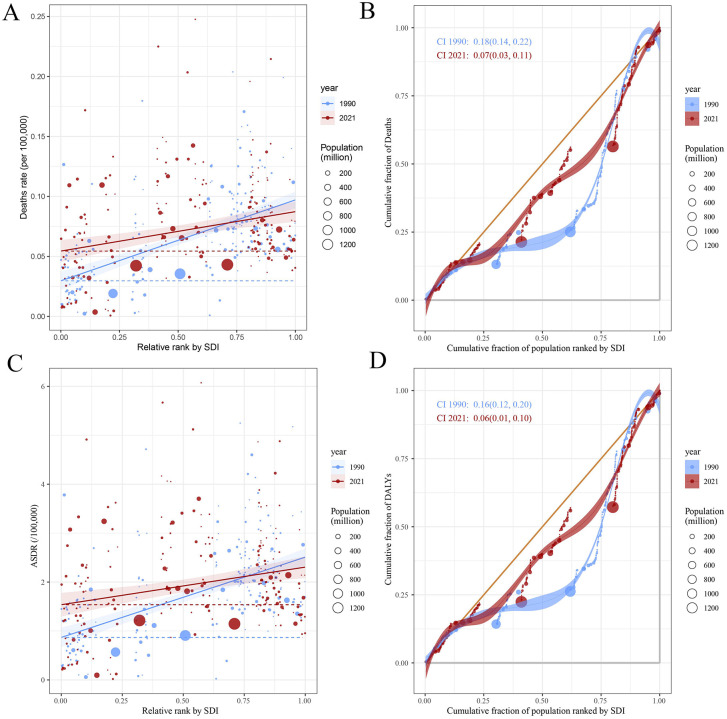
Slope indexes and concentration indexes for ASMR and ASDR of TC-HBMI from 1990 to 2021 worldwide. **(A-B)** ASMR of TC-HBMI **(C-D)** ASDR of TC-HBMI. ASMR: Age-standardized mortality rate. ASDR: Age-standardized DALY rate. TC-HBMI: Thyroid cancer attributable to high body-mass index. DALY: Disability-adjusted life year.

Continuing with the above conclusion that ASMR, ASDR and EAPC of ASMR and ASDR were highly correlated with SDI, we performed further analyses of health inequities (Fig 9). The slope index of death rate of TC-HBMI decreased slightly from 0.07(95% CI: 0.06, 0.08) in 1990 to 0.03(95% CI: 0.02, 0.05) in 2021, while the concentration index decreased from 0.18(95% CI: 0.14, 0.22) to 0.07(95% CI: 0.03, 0.11) in 2021(Fig 9A, 9B). The slope index of TC-HBMI ASDR decreased from 0.07(95% CI: 0.06, 0.08) in 1990 to 0.03(95% CI: 0.02, 0.05) in 2021, while the concentration index decreased from 0.18(95% CI: 0.14, 0.22) to 0.07(95% CI: 0.03, 0.11) in 2021(Fig 9C, 9D).

### 3.5. Frontier analysis for the association between ideal ASMR/ADMR of TC-HBMI and SDI

**Fig 10:**
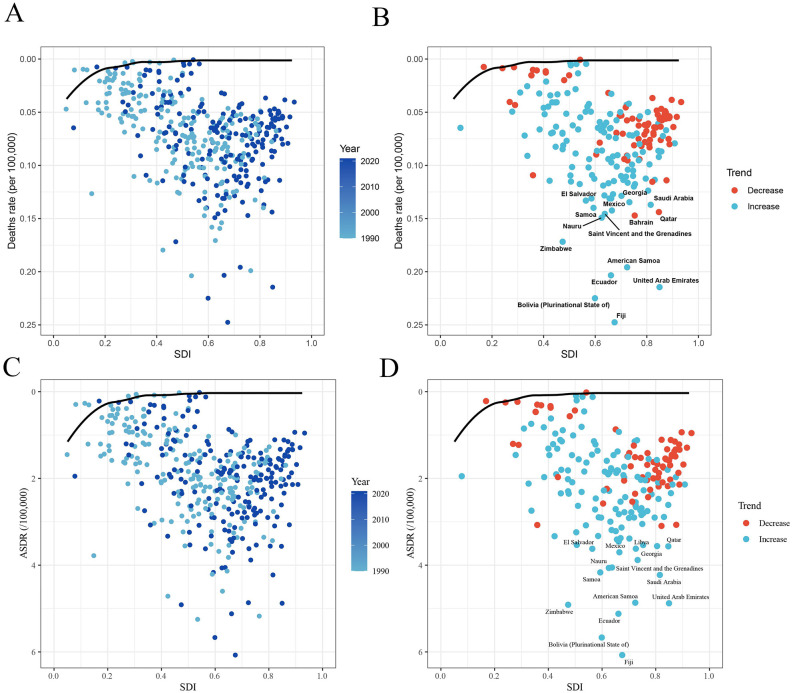
Frontier analysis based on SDI and ASMR/ASDR of TC-HBMI in 204 countries and territories. **(A-B)** ASMR of TC-HBMI, **(C-D)** ASDR of TC-HBMI. ASMR: Age-standardized mortality rate. ASDR: Age-standardized DALY rate. TC-HBMI: Thyroid cancer attributable to high body-mass index. DALY: Disability-adjusted life year. SDI: Sociodemographic Index.

In order to explore the ideal situation in which countries could control the burden of disease under the SDI conditions corresponding to each year, frontier analysis was performed (Fig 10). In the results of the frontier analysis, the 15 countries furthest from the frontier fit line in all countries are marked in black. Among the ASMR of TC-HBMI, the countries furthest from the frontier line include El Salvador, Georgia, Saudi Arabia, Mexico, Samoa, Nauru, Bahrain, Qatar, Saint Vincent and the Grenadines, Zimbabwe, American Samoa, Ecuador, United Arab Emirates, Bolivia (Plurinational State of), Fiji. For ASDR of TC-HBMI, countries furthest from the frontier line include El Salvador, Georgia, Saudi Arabia, Mexico, Libya, Samoa, Nauru, Qatar, Saint Vincent and the Grenadines, Zimbabwe, American Samoa, Ecuador, United Arab Emirates, Bolivia (Plurinational State of), Fiji.

### 3.6. Death numbers, DALY numbers, ASMR and ASDR trends predicted by Bayesian age-period-cohort (BAPC)

**Fig 11:**
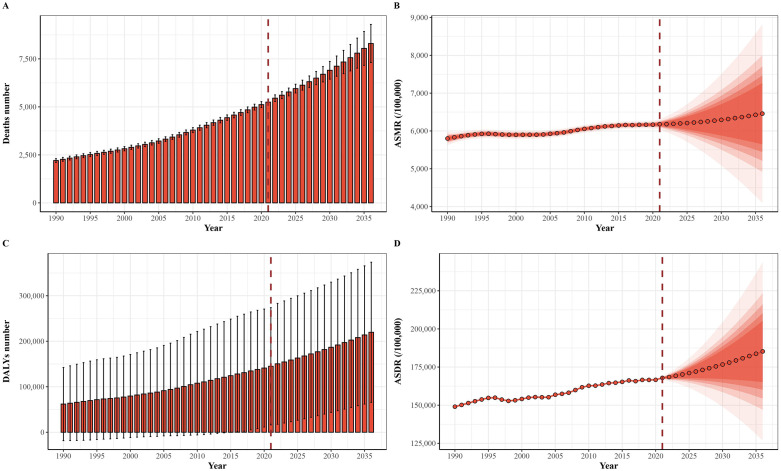
Prediction of the death and DALY rates of TC-HBMI from 2021 to 2036 in age-standardized population (A) the ASDR of TC-HBMI. **(B)** The ASDR of TC-HBMI. ASMR: Age-standardized mortality rate. ASDR: Age-standardized DALY rate. TC-HBMI: Thyroid cancer attributable to high body-mass index. DALY: Disability-adjusted life year. EAPC: estimated annual percentage change.

To forecast the trends in age-standardized mortality and DALY rates for 2036, we performed BAPC analysis. The results are summarized in Fig 11. Overall, the predictions indicate that death numbers, DALY numbers, age-standardized mortality and DALY rates are expected to rise in the coming years. Specifically, the global death/DALY numbers of TC-HBMI are projected to be approximately 8301/219711, ASMR/ASDR are projected to be approximately 0.064/ 1.852 by 2036.

## 4. Discussion

The research findings indicate a consistent global increase in the TC-HBMI between 1990 and 2021, as reflected by rising ASMR and ASDR in most regions, particularly in middle, low-middle, and low SDI regions. The continuous increase in the global burden of TC-HBMI from 1990 to 2021, as evidenced by the rising deaths and DALYs, can be attributed to several factors. A primary driver of this trend is the significant global rise in obesity prevalence during this period. The concurrence of increasing TC incidence and obesity prevalence has been noted, suggesting a potential temporal link [17]. Changes in diagnostic practices and increased detection of TC also play a role. The rise in incidence has been partly attributed to enhanced detection through screening and improved diagnostic processes, leading to the identification of more cases, including potentially indolent papillary carcinomas [22]. However, the observation of statistically significant increases in the incidence of larger-sized (>2 cm) and advanced-stage papillary TC, along with a rise in TC mortality, suggests that the escalating burden is not solely due to overdiagnosis [23]. Similarly, the increased incidence of larger tumors with aggressive histological features points towards a genuine rise in disease development, potentially influenced by increasing exposure to risk factors like obesity [24]. Therefore, while improved detection methods contribute to the higher number of diagnosed cases, the concurrent rise in obesity prevalence and the increase in more clinically significant TC indicate that the growing burden of TC-HBMI is likely a consequence of both enhanced detection and a true increase in disease incidence driven, at least in part, by the escalating rates of obesity worldwide.

Notably, the trends exhibit periods of varying growth rates and even a temporary decline, suggesting a complex interplay of factors influencing this association. The initial increase in ASMR and ASDR of TC-HMBI during 1990–1995 likely reflects the nascent stages of the global obesity epidemic [5]. Simultaneously, while advancements in diagnostic techniques for TC were underway during the early 1990s, the association between obesity and TC was beginning to be recognized in the scientific literature [25,26]. The subsequent decrease in ASMR of TC-HMBI between 1995 and 2004 presents an interesting contrast to the overall trend. Although global obesity rates continued their upward climb during this period, it is possible that the rate of increase might have temporarily slowed down in certain regions, or that there was a delayed manifestation of the cumulative effects of BMI on TC mortality and morbidity. This time frame also witnessed further progress in the diagnosis and treatment of TC [27]. Therefore, improvements in overall TC management strategies, which might not have been exclusively targeted towards obesity-related cases, could have contributed to a temporary reduction in the ASMR of TC-HMBI. The renewed increases in ASMR and ASDR of TC-HMBI from 2004 to 2013 and continuing into 2013–2021 exhibit a strong temporal correlation with the significant acceleration of the global obesity pandemic during these years. Data from various sources within the research material corroborate this trend [28]. While advancements in diagnostic and therapeutic modalities for TC continued, the increasing burden of TC-HMBI suggests that the sheer magnitude of the escalating obesity epidemic might have outweighed the impact of these improvements for this specific subtype of TC [29]. The established association between obesity and more aggressive clinicopathological features of papillary TC could also have contributed to this renewed increase in mortality and morbidity [30]. Between 1995 and 2004, a temporary decline in mortality and DALY rates attributable to high BMI was observed. This phenomenon could be explained by the lagging biological effect of BMI on thyroid carcinogenesis, together with concurrent advances in diagnostic imaging and therapeutic interventions that improved prognosis and temporarily reduced disease burden. Furthermore, enhanced data sources and methodological updates in the GBD estimation process during this time may have partially contributed to the observed fluctuation.

The finding that women experienced a greater burden of TC-HBMI suggests the involvement of sex-specific factors. Hormonal influences, particularly the role of female hormones such as estrogen, have been implicated in TC development and may interact with obesity to a greater extent in women [31]. The incidence differenng the role of hormonal factors [32]. Adipokines, hormones produced by fat tissue, also play a role and may exhibit sex-sptiated TC is markedly higher in women, especially during their reproductive years, further suof pportiecific effects in obese individuals. Women tend to have a higher percentage of adipose tissue with different distribution patterns compared to men, which could influence adipokine profiles and their impact on TC risk [33]. Lower levels of adiponectin, a potentially protective adipokine, have been observed in obese patients, particularly women with papillary TC [34].

The observed heterogeneity in ASMR and ASDR trends between gender groups across different SDI regions highlights the complex interplay of socioeconomic factors and sex-specific mechanisms in the development of TC-HBMI. In high-middle SDI regions, the increasing trend of ASMR and ASDR in males contrasts with the decreasing trend in females. Similarly, high-SDI regions show a stable trend in males and a marked decline in females for both ASMR and ASDR. This divergence suggests that the impact of high BMI on TC mortality and morbidity is evolving differently for men and women depending on the level of socioeconomic development. While globally, women have a higher incidence of TC, the faster increase in ASMR and ASDR in males in certain SDI regions 1 could indicate a narrowing of this gender gap in disease burden over time [31]. This shift might be attributed to factors such as a lower health awareness, a more pronounced increase in obesity rates and comorbidities such as cardiovascular disorders among men in these regions [31,35,36]. Conversely, the upward trends in lower SDI regions might reflect increasing obesity rates coupled with limited access to preventive and diagnostic services [37]. While the stable or decreasing trends in TC-HBMI burden among females in high and high-middle SDI regions could be due to various reasons, including better access to healthcare, earlier detection, and potentially more effective management of obesity and related risk factors in these populations [38]. The highest ASDR observed in the high SDI region in 2021, despite a downward trend in deaths and DALYs, could be influenced by higher prevalence rates and longer survival times due to advanced healthcare systems [38]. These projections are conditional on the continuation of present epidemiological and metabolic trends. Future variations in global obesity prevalence—either due to intensified prevention strategies or worsening lifestyle patterns—could substantially modify the actual burden. Incorporating dynamic BMI forecasts in future models would allow for sensitivity analyses under alternative scenarios.

The heterogeneity in ASDR and ASMR of TC-HBMI across different SDI regions in 2021 highlights the complex interplay between socioeconomic development and the burden of this disease. Notably, Andean Latin America, Central Sub-Saharan Africa, and Central Latin America exhibited markedly higher ASDR and ASMR compared to other regions with similar SDI values. This suggests that factors beyond the general SDI, such as specific dietary patterns, genetic predispositions, or environmental exposures prevalent in these regions, may contribute to a heightened risk of TC-HBMI [39–41]. Correlation analysis further revealed a significant positive correlation between both ASMR and ASDR of TC-HBMI with the SDI. This indicates that as the socioeconomic development of a region increases, the mortality and morbidity rates associated with TC-HBMI also tend to rise. This observation could be linked to higher prevalence of obesity in more developed regions, as well as increased detection rates due to better access to healthcare and diagnostic technologies. Conversely, the EAPC of ASMR/ASDR exhibited a significant negative correlation with SDI. This implies that regions with higher initial rates of mortality and morbidity experienced a greater annual increase, but this increase was more pronounced in regions with lower SDI. This could suggest that while higher SDI regions have a greater overall burden, lower SDI regions are experiencing a faster growth in this burden over time.

Decomposition analysis from 1990 to 2021 indicated that population change was the primary driver of the increase in both deaths and DALYs of TC-HBMI across all five SDI regions. Notably, in high-SDI regions, aging showed a negative contribution to TC-HBMI deaths. This finding implies that the demographic shift toward an older population did not proportionally increase obesity-related thyroid cancer mortality. The phenomenon may reflect healthier aging with better lifestyle management, widespread early diagnosis, and therapeutic improvements that have reduced fatal outcomes among elderly patients. Furthermore, older cohorts in high-SDI settings were less affected by the modern obesity epidemic, delaying the manifestation of BMI-related cancer risk. This underscores the impact of a growing global population on the overall burden of the disease. However, demographic change negatively affected deaths and DALYs in high-middle and high SDI regions, and aging also had a negative impact on TC-HBMI deaths in high SDI regions. This suggests that in more developed regions, while population growth contributes to the overall increase, factors like aging populations and shifts in age structure might be mitigating the rise in mortality and morbidity to some extent. Further analysis of health inequities revealed a modest reduction in disparities over time. These reductions suggest that global efforts to improve healthcare access and socioeconomic conditions may have contributed to narrowing health inequities, albeit modestly. However, the persistence of disparities indicates that targeted interventions remain necessary, particularly in regions with lower SDI levels where the burden of TC-HBMI remains high [42,43].

Frontier analysis provided insights into the ideal disease burden control achievable under varying SDI conditions. For instance, small island nations like Samoa, Nauru, and American Samoa may face unique barriers such as limited healthcare infrastructure and high prevalence of obesity, a key risk factor for TC-HBMI [44]. Similarly, countries like El Salvador and Bolivia may be constrained by socioeconomic instability and inadequate access to early detection and treatment services [45]. In contrast, wealthier nations like Qatar and the United Arab Emirates, despite high SDI, may experience elevated TC-HBMI burdens due to lifestyle factors associated with rapid urbanization and dietary shifts [46]. The observed reduction in the concentration index may reflect improved access to early detection tools in low-income countries, although disparities remain.

Some limitations should be considered in this study. BMI, as the primary measure of adiposity, is a crude indicator and does not differentiate between fat and muscle mass or account for body fat distribution. This could potentially lead to misclassification and an underestimation of the true association. The observational nature of most studies limits the ability to definitively establish causality, and the potential for residual confounding factors cannot be entirely ruled out. Variations in diagnostic criteria and screening practices for TC across different regions and time periods might also introduce heterogeneity in the incidence data. Additionally, specific histological subtypes of TC was not identified, making it challenging to determine if the association with BMI varies across different types of this malignancy. As with all GBD-based analyses, our findings are subject to the limitations of secondary data modeling, including potential underestimation in regions with sparse cancer registry coverage or overweight data.

## 5. Conclusion

The global burden of TC attributable to high body-mass index has significantly increased between 1990 and 2021, with women experiencing a disproportionately higher burden. The significant public health implications of these findings necessitate comprehensive strategies to address the global obesity epidemic and to consider sex-specific approaches in prevention and risk assessment. Continued research utilizing more refined measures of adiposity and exploring the underlying molecular mechanisms is crucial for developing targeted interventions to mitigate the impact of obesity on TC risk and reduce its increasing global burden.

## Supporting information

S1 Table Analysis of the GBD 2021 data reveals. (XLSX)

S2 Table The burden of age-standardized rates of DALYs and death numbers for 1990 and 2021. (XLSX)

S3 Table The greatest annual decrease in ASDR. (XLSX)

S4 Table The EAPC of ASMR and ASDR of 1990–2021. (XLSX)
